# Distinctive Pathophysiology Underlying Constipation in Parkinson’s Disease: Implications for Cognitive Inefficiency

**DOI:** 10.3390/jcm9061916

**Published:** 2020-06-19

**Authors:** Rosalind M. Tucker, Suzanne Ryan, Bu’ Hussain Hayee, Ingvar Bjarnason, Aisha D. Augustin, Chianna Umamahesan, David Taylor, Clive Weller, Sylvia M Dobbs, R John Dobbs, André Charlett

**Affiliations:** 1 Institute of Pharmaceutical Science, King’s College London, London SE1 9NH, UK; rosalind.tucker@kcl.ac.uk (R.M.T.); aisha.augustin@kcl.ac.uk (A.D.A.); chianna.umamahesan@kcl.ac.uk (C.U.); david.taylor@slam.nhs.uk (D.T.); clive.weller@kcl.ac.uk (C.W.); andre.charlett@phe.gov.uk (A.C.); 2 The Maudsley Hospital, London SE5 8AZ, UK; 3 Imaging, King’s College Hospital, London SE5 9RS, UK; suzanneryan@nhs.net; 4 Gastroenterology, King’s College Hospital NHS Foundation Trust, London SE5 9RS, UK; b.hayee@nhs.net (B.H.H.); ingvarbjarnason@mac.com (I.B.); 5 Statistics, Modelling and Economics, National Infection Service, Public Health England, London NW9 5EQ, UK

**Keywords:** Parkinson’s disease, colonic transit-time, functional constipation, cognitive-processing time, anxiety, depression

## Abstract

Depression is associated with constipation within and outside Parkinson’s disease (PD). Since inefficient cognitive-processing (bradyphrenia) features in PD and an enterokinetic agent improved cognitive performance in healthy individuals, bradyphrenia may be associated with constipation. We aim to define the archetypical bowel function of PD, and its association with cognition, mood, and motor features within and outside PD. We assessed colonic transit time (oral radio-opaque markers over 6 days), bowel function and psychometric questionnaires and measures of PD facets, including bradyphrenia, in 58 participants with diagnosed PD, and 71 without (controls). The best abdominal X-ray (day 7) predictors of PD status were total retained marker count and transverse colon segmental delay. However, Rome functional constipation status complemented segmental delay better, giving good specificity (85%) but low sensitivity (56%). Transverse colon marker count appeared to be age-associated only in PD. In PD, those correctly classified by bowel dysfunction had higher depression scores (*p* = 0.02) and longer cognitive-processing times than the misclassified (*p* = 0.05). Controls misclassified as PD by bowel dysfunction had higher depression and anxiety scores than the correctly classified (*p* = 0.002 and 0.003, respectively), but not slower cognitive processing. Measures of motor features were independent of sub-classification by bowel function in PD and in controls. In conclusion, constipation in PD has distinct localized pathophysiology, and is associated with bradyphrenia.

## 1. Introduction

The worldwide prevalence of chronic constipation in diagnosed Parkinson’s disease (PD) [1,2] is much higher than in the general population [3,4], whether self-reported or criterion-based. It is unclear whether we are dealing with a single aetiopathogenic entity with variation in progression, or with divergent or separate entities. We have characterised the natural history of constipation in relation to PD by a decreased frequency of defaecation up to three decades before the median age of neurological diagnosis, and worsening constipation thereafter [5]. Metanalysis [6] and primary care database mining [7] show that constipation doubles the risk of PD diagnosis 10 years on, and the severity of constipation increases that risk [8]. In those not labelled with PD in life, a history of lower frequency of defaecation is associated with decreased substantia nigral neuronal density *post mortem* [9] and a four-fold increase in Lewy bodies [10] (pathological hallmark of PD in brain). Moreover, in established PD, worsening of self-assessed constipation correlates with nigrostriatal dopaminergic neuronal loss on functional imaging [11].

In 1817, James Parkinson [12] implied both slow intestinal transit in PD (need for “stimulating medicines”) and disordered defaecation [13] (“expulsion of faeces from the rectum required mechanical aid”). Nearly 200 years on, prolonged orocaecal transit time, using lactulose hydrogen breath testing, was described in PD patients without small intestinal bacterial overgrowth (SIBO) [14]. More recently, using a radio-labeled meal, the beneficial effect of an enterokinetic agent on gastrointestinal and colonic transit was demonstrated in constipated PD patients without rectal evacuation disorder [15]. Our purpose, building on the smaller study of Knudsen et al. [16], is to define archetypical PD bowel function. We seek features discriminant for PD status (i.e., with or without diagnosed PD) in the abdominal X-ray taken after 6 days’ oral radio-opaque markers, complementing these by bowel function questionnaires. We stratify PD status according to correct or misclassification by bowel function, with a view to relating these subcategories to mood, cognition, and measures of cardinal signs of PD.

Depression and anxiety are pre-diagnostic features of PD, predated by constipation [7,17]. Cognitive decline is not a pre-diagnostic feature [7], but is associated with constipation after diagnosis [2]. However, the natural history of bradyphrenia, the slowing of cognitive processing in PD, characterised by difficulty shifting or developing an appropriate mental set in response to external stimuli [18,19], is unknown. To define its relationship to the archetypical bowel function of PD, we measure cognitive processing time. The methodology described [18] is not confounded by movement time, and results complement established mental test and depression scores. Similarly, to capture pre-diagnostic brady/hypokinesia (a *sine qua non* cardinal sign of PD [20]), we adopt provocative stress testing of the angular velocity of alternating movements [21], in addition to measuring mean stride length at free walking speed [22]. To capture any pre-diagnostic rigidity, we employ [23] objective measurement, replacing subjective rating. Clinical rating has sufficed to show increased incidence of tremor 10 years pre-diagnosis, and impaired balance 5 years before [7], so we rate these subjectively.

## 2. Methods

### 2.1. Participants

Consecutively recruited participants with clinically-definite idiopathic parkinsonism [24] (cross-referenced against U.K. brain bank criteria [20]) were studied at a national clinic, incorporating specialist gastrointestinal expertise, alongside proband-nominated controls without diagnosed PD.

Exclusion criteria were other specific neurological condition or psychosis; cardiovascular/respiratory symptoms during normal activities; other progressive or resolving disorders affecting physical ability or performance, or sufficient underlying incapacity to prevent assessments; concurrent therapy with potentially anti-dopaminergic drugs, or with hypnotics or sedatives; and serious pathology, such as neoplasm or inflammatory bowel disease. All gave informed consent to the study, approved by the King’s College London Research Ethics Committee. The STROBE checklist for cross-sectional studies was followed [25].

### 2.2. Assessments

Colonic transit time was assessed over a two-year period, ending May 2019, using methodology pioneered by Abrahamsson et al. [26] and subsequently modified and validated [27,28,29,30,31]. Participants were instructed to swallow a pellet (Transit-Pellets, Medifactia, Stockholm, Sweden), containing ten ring-shaped radio-opaque barium markers, at 10:00 a.m. on five consecutive days. On day 6, a pellet containing five rod-shaped markers was swallowed at 10:00 h and another at 22.00 h. (allowing rapid transit to be measured). An abdominal radiograph was taken on day 7, approximately 12 h after the last pellet. All radiographs were assessed by the same specialist consultant gastroenterological radiologist. Retained markers were counted for each ascending, transverse, descending, and sigmoid colon and for rectum. Segmental delay was defined as more than 10 markers per segment, and overall colonic transit delay by the total number of markers retained. The diameter of each segment was measured. The Leech Score for faecal loading [32] has high within-observer, between-occasions, and between-observer agreement [33], and is validated for use in adults [34]. The score (no faeces visible = 0; scanty faeces visible = 1; mild faecal loading = 2, moderate = 3 or severe = 4; or severe faecal loading with bowel dilatation = 5) was applied to each of three large bowel regions: right side and left side (defined by mid-vertebral line) and rectosigmoid.

The Rome III Classification of Functional Bowel Disorders [35] and Bristol Stool Scale [36] (on last motion passed and estimated median over last 3 months) were applied. Single aspects of bowel habit (covering transit and evacuation problems) were documented: number of bowel openings per day, number of days per fortnight when bowels opened, per cent of times when emptying satisfactory and per cent of times with straining. Any laxative or antiparkinsonian medication was recorded. Any use of opioid analgesics was actively discouraged throughout the evaluation period.

The objective measurements defining the clinical phenotype were of (i) hypokinesia (mean stride length at free walking speed [37]), (ii) bradykinesia (angular velocity (pronation/supination of hands [21] and dorsiflexion of foot)), (iii) rigidity (torque required for passive flexion/extension of forearm about the elbow [23]), (iv) reaction time (following an alerting signal, which does or does not warn whether imperative will be to break contact of right or left index finger from its touch-sensitive pad [18]), and (v) cognitive processing time (efficiency of response to that warning, as given by unwarned minus warned reaction time [18]). Stress test conditions (“as far and as fast as possible”) as *per* United Parkinson’s Disease Rating Scale (MDS-UPDRS, Part III, Motor Examination 3.6 and 3.7 [38]) were applied to angular velocity measurements. Instrumented devices were used to measure angular velocity over 20 s (order by side randomised). These were a doorknob and a lightweight, flat-plate hinged at the heel end, with the toe end of the shoe strapped to the plate. Presence/absence of rest, postural or action tremor was noted, and UPDRS rating for posture (3.13) was made by two assessors. Mini-mental questionnaire [39], Beck’s depression and anxiety inventories [40,41], and REM sleep disorder questionnaire [42] scores were obtained by a single assessor.

### 2.3. Statistical Analysis

Differences between those with and without PD in X-ray measurements were assessed using an appropriate generalised linear regression model (GLM). For radio-opaque markers, a grouped logistic model was used, with the number retained out of fixed number administered as outcome. To account for model over-dispersion, standard errors were inflated by the square root of the deviance-based dispersion. For segmental delay as a binary outcome, standard logistic regression was employed. Colonic diameter measurements were positively skewed: a GLM assuming Gaussian errors, with a logarithmic link function, was employed, giving estimates of relative geometric means for colonic diameters. For the ordinal faecal loading score, quantile regression models were used to obtain estimated difference in medians. Age was included in all models as a potential confounder. Gender, height, weight or body mass index was included where appropriate: none had clinically important associations with X-ray measurements or confounded clinical phenotype associations.

A two-stage analysis was conducted to enable support or contradiction of the hypothesis that individual associations between bowel function and clinical phenotype can be extrapolated from established PD to a pre-presentation state and *vice versa*. First, participants were classified as to PD status according to independent variables describing bowel function. A penalised logistic regression model was used to account for data separation due to sampling zeros [43]; this imposes a penalty on the likelihood, which has the effect of shrinking the model coefficients towards zero. Second, both participants with PD and without PD were sub-grouped into those correctly and incorrectly classified by bowel function, and differences in clinical phenotype between these subcategories were described.

## 3. Results

Demographic and phenotypic characteristics of the 58 participants with diagnosed PD and the 71 without are contrasted in Table 1. Demographic characteristics were similar except for medication. Phenotypic characteristics were appropriately dissimilar, apart from cognitive processing time.

### 3.1. Evaluation of X-Rays

**Figure 1:**
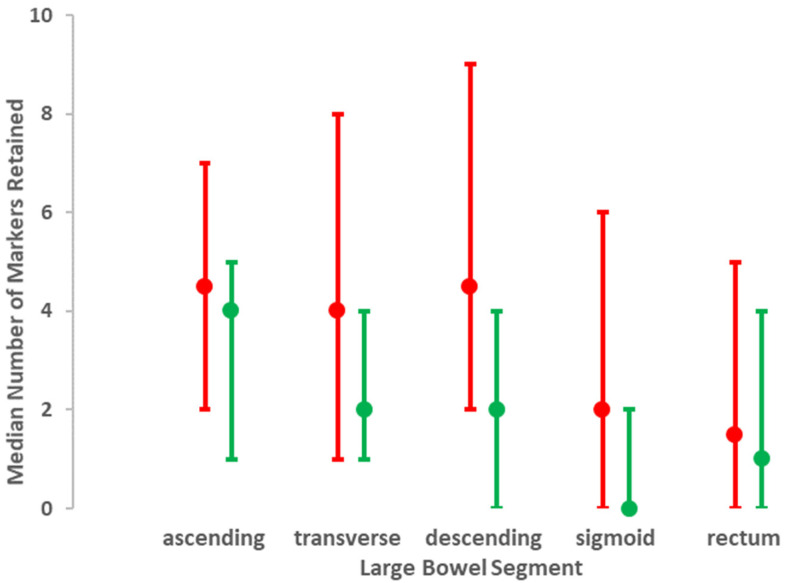
Distribution of markers retained per segment of the large bowel. The median (lower, upper quartile) for each segment is shown for those with diagnosed PD (red) and without (green).

**Figure 2:**
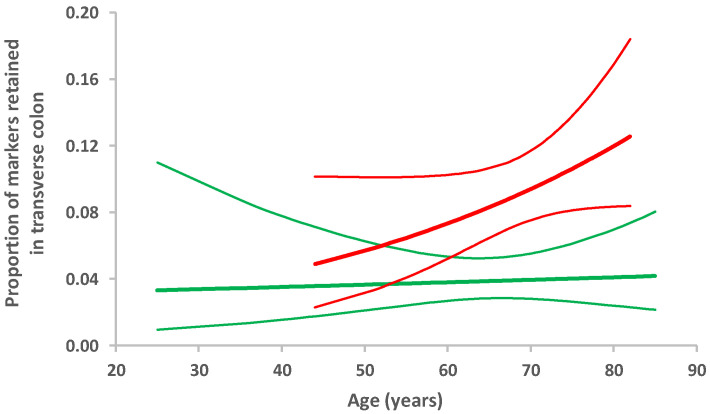
Estimated proportion of markers retained in the transverse colon by age, obtained from a generalised linear model including interaction between disease status and age. There is no evidence of any age trend in those without diagnosed PD (green), despite the wide age range: estimated odds ratio = 1.00 (95% CI: 0.97 to 1.04) per year. In contrast, in those with PD (red), there is some evidence of an increasing age trend: estimated odds ratio = 1.03 (95% CI: 1.00 to 1.06) per year. However, the interaction between PD status and age did not reach statistical significance, indicating either insufficient sample size or a chance observation.

In participants with PD, the median number of radio-opaque markers retained from the total of 60 ingested was twice that in the controls (20 (5, 95 percentile: 5, 57) *cf*. 10 (5, 95 percentile: 2, 32), respectively). Figure 1 illustrates the distribution of markers between large bowel segments for the two groups. Table 2 evaluates the effect of PD on measures taken from the X-rays. The odds of having retained markers were doubled in PD compared with the remainder for transverse, descending, and sigmoid colon, peaking in the transverse, but were not increased for rectum. Overall, any additional effect of age to that of PD status appeared small, peaking in the transverse colon. However, fitting an interaction-term between PD status and age (Figure 2) allowed us to propose that the transverse colon is susceptible to an ageing effect in PD, which is not seen in those without PD. It is noteworthy that 21% of PD probands crossed the threshold (10 markers) for segmental delay in the transverse colon, whereas none of the controls did.

**Figure 3:**
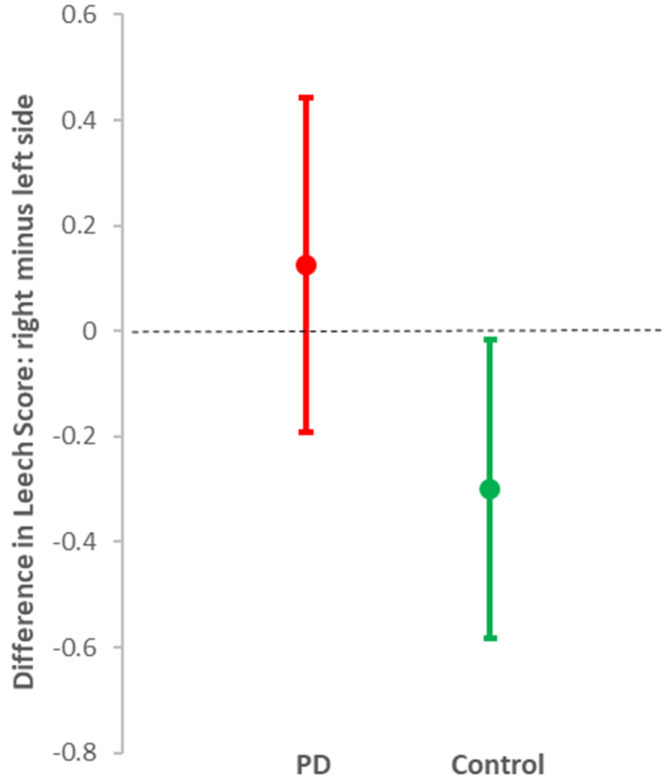
Contrast of mean (95% CI) Leech Score for faecal loading on the right side of the colon compared with left, in those with diagnosed PD (red) and controls without PD (green).

Overall, mild to moderate faecal loading was seen in those with and without PD (median total Leech Scores = 7 (5, 95 percentile: 2, 9) and 6 (5, 95 percentile: 0, 9), respectively). However, loading was significantly greater in both the right and left colon in PD than in controls, with no indication of an additional effect of ageing (Table 2). Outside PD, faecal loading was less in the right colon than in the left (mean difference in score = –0.30 (95% CI: –0.02, –0.58), *p* = 0.04), whereas in PD, there was similar loading on both sides (0.13 (95% CI: –0.19 to 0.44), *p* = 0.4) (Figure 3). This is consistent with the damming-back of faeces from a hold-up in the transverse colon in PD. The measured colonic diameter did not reflect the differential distribution of faeces by PD status. Significant gas was seen on X-ray in only three participants with PD, and one without.

### 3.2. Evaluation of Bowel Function Questionnaires

In Table 3, responses to questionnaires on bowel function are evaluated as predictors of PD status. Functional constipation status stands out as the best individual predictor.

### 3.3. Defining Archetypical PD Bowel Dysfunction

Two approaches were made to predict PD status from bowel function. For Model A (Table 4), all available measures of bowel function (i.e., features of colonic transit time, X-ray, and questionnaires) were considered. Model B was restricted to X-ray features alone. Model A classified PD status correctly in 72%, Model B performing less well, with 66% correct. Model A’s specificity (85% (95% CI: 74%, 93%)) was good, but sensitivity was low (56% (95% CI: 42%, 70%)). Model B also had good specificity (90% (95% CI: 81%, 96%)), but less sensitivity (36% (95% CI: 24%, 50%)). Whereas predictors in Model A are segmental delay in the transverse colon and functional constipation status, in Model B, the total number of markers retained replaces functional constipation status.

### 3.4. Cognition, Mood, and Motor Features in Relation to Bowel Dysfunction

**Figure 4:**
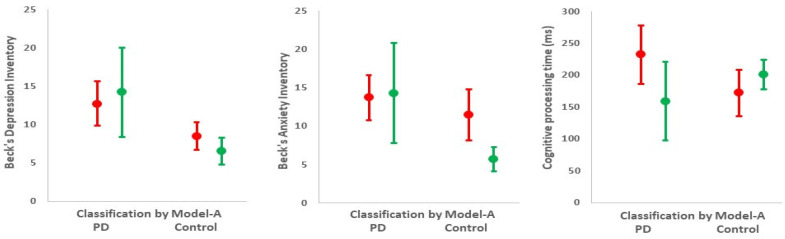
Psychometric phenotype according to subclassification by bowel function (Model A) in diagnosed PD group and in control group. Mean (95% CI) depression score, anxiety score, and cognitive processing time are shown for PD (red) and for controls (green). Of PD and control groups, 58% and 85%, respectively, were correctly allocated by bowel function, whilst 42% and 15% were allocated incorrectly.

Figure 4 compares mood and cognitive processing between those correctly classified and misclassified by bowel function using Model A. Within PD, Beck’s depression inventory score was higher in those classified correctly by bowel function than in those who were misclassified (12.7 (95% CI: 9.9, 15.6) *cf*. 8.5 (95% CI: 6.7, 10.3), *p* = 0.02). Outside PD, the depression score was higher in those misclassified as PD than in those correctly classified (14.2 (8.4, 20.0) *cf.* 6.5 (4.8, 8.3), *p* = 0.002). In PD, cognitive processing time was longer in those correctly classified (232 (186, 278) *cf*. 172 (135, 208) ms; *p* = 0.05). In apparent contrast, outside PD, processing time tended to be better in those who were misclassified as having PD (159 (95% CI: 97, 220) *cf*. 201 (95% CI: 178, 225) ms). This may, however, reflect their higher anxiety score (14.3 (7.8, 20.8) *cf*. 5.7 (4.1, 7.3); *p* = 0.003). Indeed, their anxiety was at the level characteristic of PD (where it was uninfluenced by bowel function). No other clinical phenotypic characteristic (Table 1) was associated with subclassification by bowel function.

## 4. Discussion

### 4.1. Contradictions to Single Nosological Entity Hypothesis

The picture emerging is of distinct, localized colonic pathophysiology in diagnosed PD: that is of a separate or divergent nosological entity from functional constipation. There is transverse colonic dysfunction in PD, and, probably consequent, loss of the normal pattern of less faecal loading in the right colon than in the left. Moreover, this localised dysfunction appears to be progressive with age. Lewy body pathology in the colon may also be progressive with age: it is described 2 to 5 years prior to PD diagnosis [44]. Outside PD, it is associated with clinical colitis in humans, and experimentally, with chemically-induced colitis [45]. Caeco-ileal reflux of organisms could underlie the excess of SIBO in established PD [10,46,47,48]. However, an additional provocateur to be considered is generalised gastrointestinal dysfunction of mitochondrial, metabolomic, or (local or remote) pathogen-related origin, or dysfunction consequent on involvement of vagal dorsal nuclei [49]. The similarly high frequency of SIBO reported in PD probands and their spouses (two-thirds on lactulose hydrogen breath testing [46]) could hold a solution.

Bradyphrenia is associated with the constipation of PD. In those with the archetypical PD bowel function, but without a diagnosis of PD, anxiety was at PD level and may have masked bradyphrenia. Indeed, their processing time tended to be faster, suggesting a preserved capacity to raise a “flight or fight” response to anxiety. In contrast, depression and constipation appear to be part of the same nosological entity, irrespective of PD status, and not reliant on localised colonic pathology. This nosological entity might also include SIBO, since depression (and anxiety) are reported associates of SIBO in gastroenterology outpatients [50].

Delay in the transverse colon in PD is a robust finding, not obscured by use of laxatives in 70% of the current PD probands and in 22% of the Knudsen et al. cohort [16]. The colonic diameter, even in the transverse segment, was not a predictor of PD status in the current study. Increased volume of the transverse and rectosigmoid colon was seen in the latter study [16] and correlated with number of markers retained. Thus, the use of laxatives may confound any association between colonic transit time and measures of the cardinal signs. Having reported the year-on-year increase in objectively measured rigidity in PD being stemmed following the introduction of maintenance laxatives [23], we anticipated rigidity being associated with archetypical PD bowel function. It appears that the relationship was lost by normalising colonic volume. A global PD motor score is a blunt instrument for detecting selective change in one aspect, like rigidity: it has no association with total number of markers retained or colonic volume [16], but was worse with functional constipation in a large study where no reference was made to laxative usage [2]. However, we also found no relationship between archetypical PD bowel function and objectively measured brady/hypokinesia (even with provocative stress-testing), subjectively assessed tremor or postural abnormality.

### 4.2. Mechanistic Considerations

Neuroinflammation is an early feature of PD in the brain, and peripheral immune/inflammatory markers, serum cortisol and tumour necrosis factor-alpha have been related to facets of diagnosed PD [51]. There are gradients of global motor scores on peripheral blood mononuclear cell cytokine production and nuclear factor-kappa B expression. Cortisol is elevated by, on average, 17% in PD. Serum interleukin-6 increases with age, with elevation in PD equivalent to 10 years of ageing. Moreover, a higher concentration is predictive of incident PD 4 years on.

We discuss mechanisms by which the gut may be the source of the systemic immune activation in PD and depression. Dysbiosis, whether as SIBO, changed microbiota in the stagnant proximal colon, or both, is a potential driver. Small intestine bacterial overgrowth is found in between a quarter and two-thirds of PD probands, using a lactulose hydrogen breath test [10,46,47,48]. In PD, SIBO is associated with higher circulating natural killer (NK) and CD4+ lymphocyte counts, as well as with lower neutrophil counts [46]. Facets of PD have directionally similar associations with leucocyte subsets: rigidity and hypokinesia with higher NK and CD4+ counts (the latter apparently modulating the NK-effect on rigidity), and tremor with lower neutrophil count (probably reflecting sequestration in the gut). Our early experience of antimicrobial treatment of SIBO in PD is of, at most, a temporary effect on breath hydrogen and no sustained clinical benefit. Others have reported failure of rifaximin treatment for SIBO in over half of patients at 6 months [47]. That successive antimicrobial courses for any indication in PD were associated with cumulative increase in rigidity [37] is consistent with adverse modification of gut microbiota. Consistent with beneficial modification is maintenance laxatives stemming a long-term increase in rigidity over time [23].

Another candidate driver is colitis. Chronic colitis, with CD4+ cell mucosal infiltration, was found in 85% of PD patients with constipation [52]. Messenger RNA expression of pro-inflammatory cytokines and glial markers were elevated in ascending colon biopsies from PD patients compared with controls [53]. Faecal calprotectin (released by neutrophils upon activation), zonulin (regulates intestinal permeability by modulating tight junction function), and alpha-1-antitrypsin (reflects loss of proteins into intestinal lumen by mucosal barrier disruption) were elevated in PD compared with controls [54]. There seems to be some reversibility in both gut permeability and the systemic immune response, at least in a setting of constipation alone [55]. They returned towards normal three months after one month’s bisacodyl treatment, despite recurrent constipation. Depression and anxiety outside PD have also been linked to intestinal mucosal integrity (using plasma zonulin, intestinal fatty acid binding protein-2, and lipopolysaccharide endotoxin) [56], but reversibility has not been tested. Not only is the presence of colitis usual in PD, but also there is more PD than expected in inflammatory bowel disease (IBD). The risk ratio for PD (1.4) is similar in Crohn’s disease and ulcerative colitis [57]. Crohn’s disease and PD share two genetic risk factors for inflammation: LRRK2 risk alleles [58] and a NOD2 mutation in a protein encoded by CARD15 [59,60]. LRRK2 deficiency leads to aberrant activation of macrophages and increased susceptibility to chemically-induced colitis in mice [61]. NOD2 has a central role in controlling inflammation by maintaining equilibrium between intestinal microbiota, mucosa, and host immune responses, which is lost with mutations [62].

Vitamin D may be a player in the loss of barrier function, and, consequently, of immune homeostasis [63,64]. It acts as a modulator of innate and adaptive immune responses. People with chronic constipation and slow orocaecal and/or colonic transit [64], PD [65], or depression [63] have significantly lower vitamin D concentrations. In IBD, lower concentrations are a biomarker of disease activity and predict poor clinical outcome [66]. There are vitamin D receptors on gut mucosal cells, and in areas of the brain implicated in depression, including promotor regions of serotonin genes.

### 4.3. Study Limitations and Directions for Future Research

A limitation to the study of bradyphrenia is that only one level of complexity in cognitive processing is examined: the ability to adapt by incorporating an item of new information. There are implications for the impaired sequencing and multitasking of PD, for which further psychometric tests (similarly not confounded by movement time) are required. In study design, excluding laxative usage in both PD (anti--parkinsonian-treatment naïve) and control cohorts should allow a better grasp of the relationship of archetypical PD bowel function to measures of cardinal signs and psychological associates. A comparator group recruited for chronic constipation would, of course, come already confounded by laxative usage. Larger cohort sizes would allow verification that the proposed age-association of transverse colon dysfunction is confined to PD. Longitudinal follow-up could capture and monitor divergencies in relation to gastrointestinal pathophysiology.

Improving transverse colon transit may be disease-modifying, colonic transit time screening a focus for early intervention. For example, in a placebo-controlled, double-blind, randomized trial, a single dose (1 mg) of the enterokinetic agent, prucalopride, had a pro-cognitive effect in healthy volunteers across three separate tasks (verbal learning, incidental emotional memory, and symbol association with reward) [67]. No antidepressant profile was suggested by measures of emotional processing. This neglected avenue for maintenance intervention requires throughgoing investigation.

## 5. Conclusions

Parkinson’s disease has been described as premature ageing [68,69], and this appears to include transverse colon function. Different facets of PD may have different, not necessarily co-incident, drivers and mediators. Since constipation is the first clinical feature in the natural history of PD, it is rational to try to associate other downstream events with it, as if there were a common driver or mechanistic cascade. Our detailed assessment shows depression and constipation as close-knit entities, irrespective of the presence/absence of PD, pointing to shared drivers and mediators. We show that bradyphrenia is also constipation-linked, but delayed until PD is overt: that is bradyphrenia is driven by sequential processes.

The profile of the gut’s inflammatory response, its integrity and permeability, translocation across gut/blood barrier, and potential mediators (faecal metabolome and systemic immune response) may, in part, explain a prodrome extending back decades, different admixtures of facets at presentation, and their differential progression. Focus on improving gastrointestinal transit to lessen underlying dysbiosis is a ready-made, but practically challenging, way forward.

## Figures and Tables

**Table 1:** Participant characteristics at time of colonic transit time test X-ray.

Characteristic		Mean (95% Data Interval) ^*^		Mean Difference ^*^ (95% CI)
		PD *n* = 58	Control *n* = 71	
**Demographic**	**Age (years)**	67.6 (50.2, 85.0)	64.8 (43.6, 86.0)	2.9 (–0.7, 6.3)
	Gender (male)	58.62 ^a^	42.25 ^a^	–0.16 (–0.33, 0.01) ^c^
	Height (cm)	169.1 (148.8, 189.5)	171.2 (151.4, 191.0)	–2.1 (–5.7, 1.5)
	Weight (kg)	70.9 (42.9, 98.9)	73.6 (45.1, 102.2)	–2.7 (–7.8, 2.3)
	Time since diagnosis (years)	5 (0, 21) ^b^	na	na
	Laxatives ^†^ (% taking)	69.1 ^a^	14.5 ^a^	0.55 (0.40, 0.69) ^c^
	Anti-Parkinsonian medication ^ⱡ^ (% taking)	83.0 ^a^	na	na
**Phenotype**	Mini-mental score	30 (17, 30) ^b^	30 (28, 30) ^b^	–0.33 (–0.25, –0.41) ^d^
	Depression score	10 (1, 29) ^b^	5 (0, 27) ^b^	5 (3, 4) ^e^
	Anxiety score	11 (0, 30) ^b^	5 (0, 28) ^b^	6 (3, 8) ^e^
	REM score	4 (0, 10) ^b^	2 (0, 9) ^b^	2 (1, 3) ^e^
	Mean stride length (mm)	1118 (701, 1535)	1306 (897, 1733)	–187 (–251, –124)
	Angular velocity of forearm inwards (°/s)	525 (132, 919)	802 (455, 1149)	–276 (–343, –208)
	Angular velocity of forearm outwards (°/s)	501 (179, 823)	752 (443, 1061)	–251 (–308, –193)
	Angular velocity of foot upwards (°/s)	32 (–7, 70)	54 (5, 104)	–23 (–31, –15)
	Angular velocity of foot downwards (°/s)	46 (–7, 99)	73 (7, 138)	–27 (–37, –16)
	Flexor rigidity § on worse side (Nm·10^−3^)	482 (71, 1367) ^b^	240 (5, 931) ^b^	256 (114, 399) ^e^
	Posture by UPDRS 3.13 subscore	1 (0, 4) ^b^	0 (0, 1) ^b^	1 (0.79, 1.21) ^e^
	Tremor (% with)	64.29 ^a^	15.49 ^a^	0.49 (0.34, 0.64) ^c^
	Unwarned reaction time (ms)	680 (459, 1446) ^b^	610 (410, 841) ^b^	71 (12, 130) ^e^
	Warned reaction time (ms)	480 (208, 1192) ^b^	399 (221, 776) ^b^	81 (13, 148) ^e^
	Cognitive processing time (ms)	209 (7, 458) ^b^	204 (–14, 406) ^b^	8 (–32, 48) ^e^

* [M1] [SD2] exceptions denoted by superscript letter: ^a^ percentage; ^b^ 50th (2.5, 97.5) percentile; ^c^ mean difference in proportion (95% CI); ^d^ probability that a randomly drawn observation from those with PD is higher than one from remainder; ^e^ quantile regression. ^†^ taking laxatives either daily, almost daily, or as required. Confining this to daily or almost so reduced percentages to 56.4% for PD and 7.3% for the remainder. ^ⱡ^ anti-Parkinsonian medication used: amantadine, cabergoline, levodopa combination, pramipexole, selegiline, rasagiline, and trihexyphenidyl (low dose). Levodopa was combined with extracerebral dopa-decarboxylase inhibitor ± catechol-O-methyl transferase inhibitor, as evenly spaced as practicable to avoid iatrogenic fluctuations in performance. None were receiving a levodopa combination as monotherapy. § torque required for passive extension of forearm about elbow.

**Table 2:** Evaluation of the features of colonic transit time test X-rays, and the additional effect of age, in predicting disease status.

Characteristic	Effect of PD ^*^		Additional Effect of Age ^†^	
	Odds Ratio (95% CI)	*p*-Value	Odds Ratio (95% CI)	*p*-Value
**Number of markers retained**				
right colon	1.36 (0.99, 1.87)	0.06	1.01 (1.00, 1.03)	0.2
transverse colon	2.37 (1.58, 3.56)	0.001	1.02 (1.00, 1.04)	0.1
descending colon	2.02 (1.30, 3.13)	0.002	1.01 (0.99, 1.03)	0.4
sigmoid	2.03 (1.22, 3.39)	0.007	1.01 (0.98, 1.04) ^a^	0.5
rectum	1.18 (0.75, 1.86)	0.5	1.010 (0.99, 1.04)	0.4
Total	2.12 (1.46, 3.06)	0.001	1.02 (1.00, 1.04) ^a^	0.1
**Segmental delay (presence/absence)**				
right colon	7.17 (0.83, 62.13)	0.07	1.08 (1.00, 1.21)	0.2
transverse colon	^ⱡ^		1.04 (0.96, 1.12)	0.4
descending colon	3.39 (0.99, 11.54)	0.05	1.01 (0.95, 1.08)	0.7
sigmoid	3.59 (0.90, 14.39)	0.07	1.00 (0.94, 1.07)	0.9
rectum	1.31 (0.32, 5.33)	0.7	1.13 (1.01, 1.26)	0.03
any	4.92 (2.01, 12.05)	0.001	1.07 (1.02, 1.13)	0.01
	**Relative mean** **(95% CI)**	***p*-value**	**Relative mean** **(95% CI)**	***p*-value**
**Diameter (cm)**				
right colon	0.92 (0.79, 1.07)	0.3	1.00 (0.79, 1.07) ^b^	0.05
transverse colon	1.17 (0.97, 1.41)	0.1	1.01 (1.00, 1.02)	0.07
descending colon	1.21 (0.97, 1.50)	0.09	1.00 (0.99, 1.02)	0.6
sigmoid	1.13 (0.82, 1.55)	0.5	1.02 (0.99, 1.04)	0.2
rectum	1.28 (0.85, 1.92)	0.2	1.00 (0.98, 1.02)	0.8
	**Difference in medians (95% CI)**	***p*-value**	**Difference in medians (95% CI)**	***p*-value**
**Leech Sscore for faecal loading**				
right side colon	1 (0.28, 1.72)	0.007	0 (−0.04, 0.04)	1
left side colon	1 (0.54, 1.46)	0.001	0 (−0.02, 0.02)	1
rectosigmoid colon	0 (−0.76, 0.76)	1	0 (−0.04, 0.04)	1
total	1 (0.25, 2.25)	0.1	0 (−0.06, 0.06)	1

* see Statistical Analysis; ^†^ pooled odds ratio, across all ages, for any additional effect of age. No additional effect of gender, height, weight, or body mass index seen, with the exception of ^a^ gender for number of markers retained (odds greater in females); and ^b^ height for right colon diameter (diameter greater with height). ^ⱡ^ predicted perfectly in that all those with segmental delay in transverse colon had PD.

**Table 3:** Evaluation of responses to questionnaires on bowel function in predicting PD status.

Characteristic	Effect of PD	
	Odds Ratio (95% CI)	*p*-Value
**Rome III Functional bowel disorders**		
C1. Irritable bowel syndrome	0.59 (0.18, 1.74),	0.3
C2. Functional bloating	1.78 (0.80, 3.95)	0.2
C3. Functional constipation	4.41 (1.87, 10.40)	0.001
C4. Functional diarrhoea	1.58 (0.40, 6.17)	0.5
**Bristol stool scale**		
Last motion	0.96 (0.75, 1.23)	0.7
Median over last 3 months	1.09 (0.78, 1.51)	0.6
**Single aspects**		
Number of times bowels opened/day ^†^	1.13 (0.75, 1.72)	0.6
Number of days bowels opened/fortnight	0.83 (0.72, 0.96)	0.01
Straining (% of days when bowels opened) ^†^	1.02 (1.00, 1.03)	0.02
Satisfactory emptying (% of days when bowels opened) ^†^	0.99 (0.97, 1.00)	0.01

^†^ over last 3 months.

**Table 4:** Models for predicting PD status from bowel function.

Predictors	Odds Ratio (95% CI)	*p*-Value
**Model A: from all available measures**		
Segmental delay in transverse colon (1 = yes, 0 = no)	38.67 (2.17, 689.73)	0.01
Rome III: functional constipation	4.57 (1.89, 11.04)	0.001
Constant	0.43 (0.27, 0.68)	0.001
**Model B: from colonic transit time test X-ray**		
Segmental delay in transverse colon (1 = yes, 0 = no)	17.69 (0.96, 327.04)	0.05
Total number of markers retained	1.04 (1.01, 1.07)	0.02
Constant	0.35 0.19, 0.66)	0.001
